# BPSS: a Nextflow pipeline for Bacterial Peptide Sequence Selection to detect protein diversity

**DOI:** 10.1093/bioinformatics/btaf677

**Published:** 2025-12-23

**Authors:** Sylvère Bastien, Pauline François, Sara Moussadeq, Jérôme Lemoine, Karen Moreau, François Vandenesch

**Affiliations:** CIRI, Center for Integrative Research in Infectious Diseases and Immunology, Université de Lyon, Inserm U1111, Université Claude Bernard Lyon 1, CNRS UMR5308, ENS de Lyon, Lyon, 69007, France; Institut des Agents Infectieux, Centre National de Référence des Staphylocoques, Hospices Civils de Lyon, Lyon, 69004, France; CIRI, Center for Integrative Research in Infectious Diseases and Immunology, Université de Lyon, Inserm U1111, Université Claude Bernard Lyon 1, CNRS UMR5308, ENS de Lyon, Lyon, 69007, France; Institut des Agents Infectieux, Centre National de Référence des Staphylocoques, Hospices Civils de Lyon, Lyon, 69004, France; CIRI, Center for Integrative Research in Infectious Diseases and Immunology, Université de Lyon, Inserm U1111, Université Claude Bernard Lyon 1, CNRS UMR5308, ENS de Lyon, Lyon, 69007, France; Institut des Agents Infectieux, Centre National de Référence des Staphylocoques, Hospices Civils de Lyon, Lyon, 69004, France; Université de Lyon, CNRS, Université Claude Bernard Lyon 1, Institut des Sciences Analytiques, UMR 5280, Villeurbanne, 69100, France; CIRI, Center for Integrative Research in Infectious Diseases and Immunology, Université de Lyon, Inserm U1111, Université Claude Bernard Lyon 1, CNRS UMR5308, ENS de Lyon, Lyon, 69007, France; CIRI, Center for Integrative Research in Infectious Diseases and Immunology, Université de Lyon, Inserm U1111, Université Claude Bernard Lyon 1, CNRS UMR5308, ENS de Lyon, Lyon, 69007, France; Institut des Agents Infectieux, Centre National de Référence des Staphylocoques, Hospices Civils de Lyon, Lyon, 69004, France

## Abstract

**Motivation:**

Sequence variability can be extremely high, particularly in bacteria due to the rapid accumulation of mutations linked to their high replication rate and environmental selection pressure, which often favors diversifying selection. For most species, there are no automated, computationally efficient tools available for constructing a nonredundant database covering the allelic variability of target proteins.

**Results:**

We have thus developed Bacterial Peptide Sequence Selection, a Nextflow pipeline to define a minimal list of peptide sequences for detecting all variants of a protein of interest.

**Availability and implementation:**

All the code and containers used are freely available on Gitlab from https://gitbio.ens-lyon.fr/ciri/stapath/bpss or on Zenodo (10.5281/zenodo.16894981) under GPLv3 open-source license and DockerHub platform from https://hub.docker.com/u/stapath.

## 1 Introduction

Bacterial proteomics has undergone a major development with the appearance of a multitude of techniques ranging from gel-based (2DE, 2-DIGE) to high-throughput methods using liquid chromatography-mass spectrometry (LC-MS) instruments (Pérez-Llarena and Bou 2016). We have recently developed a targeted proteomics approach for the quantification of highly multiplexed proteins in a large collection of samples. This method consists in quantifying a list of peptide substitutes derived from the enzymatic digestion of bacterial proteins (Pivard *et al.* 2023). When setting up this method, we were faced with the problem of the allelic diversity of bacterial factors and our ability to detect all allelic variants. It was therefore necessary to use curated databases referencing protein diversity. Over the past 20 years, the number of sequences from whole genome sequencing (WGS) projects accessible via NCBI (Sayers *et al.* 2022) or EBI (Kanz *et al.* 2005) has grown steadily. Although there are certain biases, notably from a geographical point of view due to the cost of sequencing, the fact remains that these data reflect the greatest global diversity available today (Petit and Read 2018). This wealth of data has generated bacterial genomic databases listing allelic variants of specific genes, such as VFDB (Chen *et al.* 2005) for bacterial virulence factors, ResFinder (Florensa *et al.* 2022) for antibiotic resistance genes, and PubMLST (Jolley *et al.* 2018) for multi-locus sequence typing. PanKB (Sun *et al.* 2025), an interactive microbial pangenome knowledgebase, was recently developed to catalog genetic variability across microbial species. However, its coverage remains limited, with only Escherichia coli represented among the six highly virulent and antibiotic-resistant bacterial pathogens, known as ESKAPE (Miller and Arias 2024) pathogens. To overcome this limitation, WhatsGNU (Moustafa and Planet 2020), a comprehensive approach compiling the full genomic diversity of a given species into a compact database reflecting its overall variability has been developed. WhatsGNU calculates the frequency (GNU score) of a specific protein relative to the compact database and can also provide information about the orthology of each allele. To this end, sequence homology analyses are computed. However, depending on the accuracy of the user-provided database, the tools used [e.g. blastp (Camacho *et al.* 2009)], and the threshold parameters used, these analyses can introduce errors. Moreover, there is currently no automatic method for defining proteomic motifs detectable by mass spectrometry and specific to all allelic variants, whatever the bacterial species.

Here, we present Bacterial Peptide Sequence Selection (BPSS), a Nextflow (Di Tommaso *et al.* 2017) pipeline generating a nonredundant set of allelic variants (i.e. panallelome) contained in a protein database. The aim is to select a minimal list of peptide sequences encompassing all variants of the set from a user-defined reference sequence. This list of peptide sequences can then be used in genomic approaches to accurately detect the presence or absence of proteins as well as in targeted proteomic approaches to detect and/or quantify their expressions. The entire workflow was containerized either via Docker or Singularity improving reproducibility and reliability, promoting effortless deployment and usability. A flowchart summarizing the steps of the pipeline is available at: https://gitbio.ens-lyon.fr/ciri/stapath/bpss.

## 2 Methods

### 2.1 Inputs

Two inputs are mandatory for the execution of this pipeline. It expects a reference protein sequence for each of the proteins requiring peptide sequences to be found in a FASTA Amino Acid (FAA) format and a directory containing FAA files of translated coding genes from genomes. Two optional FAA files can also be used to include or exclude specific peptide sequences defined by the user.

### 2.2 Database preparation

Database preparation is carried out to check the files (Step 1), establish a list of peptide sequences found in multiple copies (Step 2), and generate a database of cleaned nonredundant proteins (Step 3). Of note, the database preparation process can be skipped by using the output files from a previous use of BPSS. It should be noted that Steps 1 and 2 are optional.

1. Each FAA file is screened to eliminate spurious genomes containing either ambiguous amino acids [J for (iso)leucine, B for asparagine/aspartate, Z for glutamine/glutamate] and characters not corresponding to specific amino acids (−, X), or a number of protein sequences differing by >15% from the median in order to eliminate outlier genomes.
2. Enzyme cleavage is then simulated to break down the protein sequences into a set of small peptide sequences. This simulation of protease-induced cleavage sites on amino acid sequences is performed by RapidPeptidesGenerator, i.e. RPG (Maillet 2020). By default, the enzyme used is a 47th enzyme added to the program, called “Trypsin-2rules,” whose rules are to cut the protein sequences after an arginine (K) or lysine (L), except when a proline (P) is located directly after either of these two amino acids. This step allows for the identification of a list of nonspecific and/or repeated peptide sequences for each genome. If the same peptide sequences are found in different genomes, they are considered multi-copy sequences; the list of multi-copy peptide sequences will subsequently be excluded when selecting peptide sequences for the final list.
3. The nonredundant database is then generated incrementally via a clustering method, using identity and coverage thresholds of 100% on sets of 10 million proteins (default) from checked FAA files (step B1). The clustering is performed by subdividing each part according to the length of the protein sequences. This makes it possible to compact several sets of proteins in parallel, which is faster than using CD-HIT (Fu *et al.* 2012) with a 100% sequence identity threshold, while limiting memory consumption. At the end of the clustering process, two files are created: a FAA format file containing the representative nonredundant protein sequences and another file containing the list of protein sequence identifiers identical to each representative protein sequence. The representative protein sequence identifier and description in the FAA format file ultimately correspond to the most redundant description found in the second file. Finally, this FAA format compacted database is then transformed into a BLAST database via makeblastdb from ncbi-blast+ v2.7.1 (Camacho *et al.* 2009), providing the nonredundant database.

### 2.3 Protein selection

The aim of this second part is to quickly obtain a subset of allelic variants from a user-defined protein reference sequence on the previously prepared nonredundant database.

(4) The search for allelic variants begins with a local alignment using PLAST v2.3.2 (Van Nguyen and Lavenier 2009), which retrieves all hits per query and all high-scoring segment pairs per hit with an expectation value set to 10 000 from the database divided into chunks of 5 million bytes by default. These results are then filtered using alignment identity and coverage thresholds set at 60%. Only the best result per genome is retained and the complete sequence is extracted from the database.
(5) Each of these sequences is then globally aligned to the reference protein sequence via the needle software included in the EMBOSS v6.6.0 tool suite (Rice *et al.* 2000). A metric corresponding to the difference in absolute value between the percentage length per pair of sequences in the global alignment and the percentage identity is used to filter out sequences that are too divergent. The identifiers of these sequences, as well as those contained in the clusters of these sequences when the database was created (step B3), are stored in a file. As long as several genome names appear, meaning that there are several sequences of the same genome, the selected allelic variants are clustered via CD-HIT v4.6.8 (Fu *et al.* 2012) using an initial identity threshold set at 70% and incremented by 0.5% each time. Only those allelic variants clustered with the reference protein sequence are retained.
(6) An additional filtering step on variant frequency is performed using a user-defined parameter; the latter is set to 98% by default, enabling 98% of allelic variants to be retained in the original database. This can be achieved by using the file listing the identifiers of sequences identical to each representative protein sequence to obtain a number of observations for each allelic variant.

### 2.4 Peptide sequence selection

This third part generates a minimum list of peptide sequences from the allelic variants retained previously. This minimum list must cover, if possible, each variant with a number of peptide sequences determined by the user (3 by default).

(7) As for database preparation (Step 1), enzyme cleavage is simulated in silico on the subset of allelic variants passing all the previous filters (Step 6). This process generates a list of potential peptide sequences to be used to quantify all these allelic variants. From the peptide sequences and allelic variants, a peptide sequence presence/absence matrix is computed. Peptide sequences considered as nonspecific (i.e. common to different protein sequences) and/or repeated during the generation of the nonredundant database (Step 2) are eliminated from this matrix. Other peptide sequences can be additionally excluded based on a user-defined list.
(8) The minimum list of peptide sequences can be first constituted by an optional file supplied by the user if the sequences are not nonspecific and are present in the matrix. In that case, the peptide sequences are removed from the matrix.
(9) A numerical list is initialized by computing the number of peptide sequences included in each allelic variant. As long as each allelic variant is not covered by at least three peptide sequences (default), the algorithm selects the allelic variants with the lowest coverage value (a). The algorithm lists the potential peptide sequences (b) contained in (a) via the peptide sequence presence/absence matrix (Step 7 or 8). From (b), the algorithm selects the peptide sequences that cover the highest number of under-covered allelic variants (<3 default peptide sequences) (c). For each of the peptide sequences in (c), the algorithm determines the list of allelic variants containing this sequence that are not sufficiently covered (d). From the list (d), the algorithm calculates the total number of potential peptide sequences (e) contained in each allelic variant via the matrix. The peptide sequence selected is the one with the lowest value in the list (e). This peptide sequence is moved from the potential list to the minimum list and is eliminated from the presence/absence matrix. The number of peptide sequences covering each allelic variant is then recalculated and the process is repeated until all variants are covered, if possible.

### 2.5 Peptide sequence validation

This last part validates the specificity of the selected peptide sequences. The minimum list of peptide sequences is aligned using blastp-short of BLAST+ v2.15.0 (Camacho *et al.* 2009) on the nonredundant database (Step 3). Results are filtered for mismatches and gaps and checked for the presence of trypsin hydrolysis-induced cleavage in silico. All protein sequences containing at least one peptide sequence are extracted from the nonredundant database. A multiple sequence alignment including the reference protein sequence is produced using Clustal Omega v1.2.4 (Sievers *et al.* 2011) in order to generate rapidly a phylogenetic tree using FastTree v2.1.11 (Price *et al.* 2010). Each of these protein sequences is also globally aligned to the reference protein sequence via the needle (Rice *et al.* 2000) software. All these steps allow to compare protein sequences containing peptide sequences to determine whether or not they correspond to the allelic diversity of the same protein. If these protein sequences correspond to variants of different proteins, such as accessory proteins never found in the same genome, these results can indicate a peptide sequence number threshold for detecting and quantifying variants of the protein of interest.

### 2.6 Outputs

BPSS produces two HTML files in the specified results folder: (i) a report from a Rmarkdown script which includes the minimum peptide sequence list as well as interactive tables and graphics for each stage of the pipeline and some details summarizing the pipeline execution, software versions, and R packages (Charif and Lobry 2007, Pagès *et al*. 2024, Yu *et al*. 2017, R Development Core Team 2024, Paradis and Schliep 2019, Wickham *et al.* 2019a, Wickham *et al*. 2016, Sievert 2020, Xie *et al.* 2024, Barnier 2024, Wilke 2024, Barrett *et al.* 2024, Chang *et al.* 2024), and (ii) a file corresponding to the pipeline visualization (directed acyclic graph) produced by Nextflow. BPSS produces also four files correspond to the database compacting steps: (i) a file summarizing the contents of the protein and multi-copy peptide sequences per genome (Step 1), (ii) a file in FAA format containing all multi-copy peptide sequences (Step 2), (iii) a file in FAA format containing all representative protein sequences (Step 3), and (iv) a file summarizing information related to each representative sequence (sequence length, representative sequence identifier, number of identical sequences, all sequence identifiers in the cluster; Step 3).

## 3 Results and discussion

**Figure 1.**
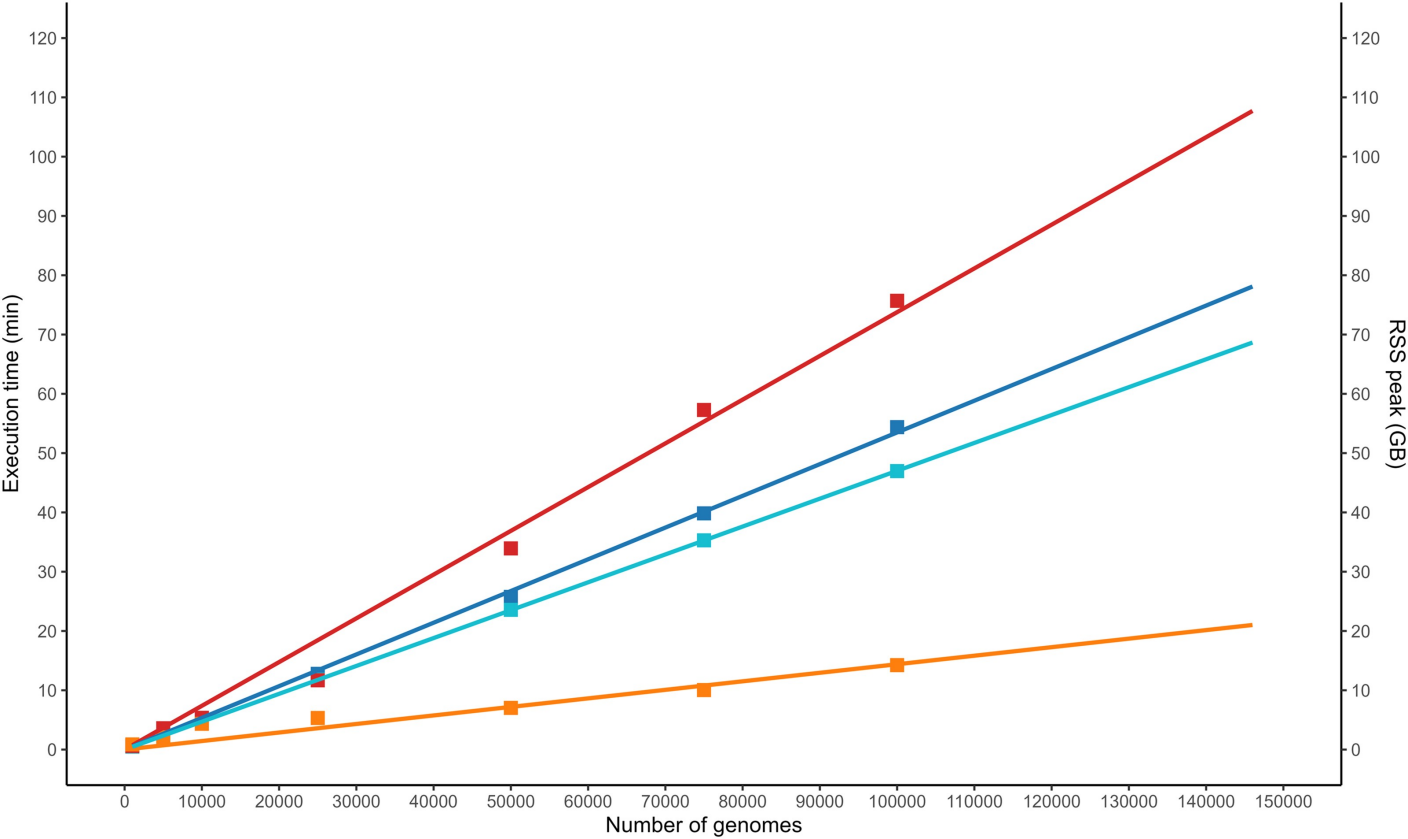
Performance metrics (execution time and RSS) of BPSS and WhatsGNU assessed across datasets containing 1000; 5000; 10 000; 25 000; 50 000; 75 000; and 100 000 *Staphylococcus aureus* genomes during the database compaction stage. BPSS execution time is represented in red and its peak resident set size in orange, while for WhatsGNU, these metrics are shown in blue and cyan, respectively.

This workflow was designed to generate a compact, nonredundant protein database while preserving the full genomic variability of the input data, all within reasonable computational time and memory consumption. To evaluate the compaction step, which is the most time-consuming, we compared BPSS with WhatsGNU (Moustafa and Planet 2020); the latter was encapsulated in a Docker container and integrated into a standardized Nextflow (Di Tommaso *et al.* 2017) pipeline. Performance metrics such as execution time, resident set size (RSS) reflecting memory consumption, and final database size were assessed across datasets containing 1000; 5000; 10 000; 25 000; 50 000; 75 000; and 100 000 *Staphylococcus aureus* genomes (Table 1, available as supplementary data at *Bioinformatics* online). To ensure a fair comparison, options for automatically removing spurious proteomes and for filtering out multi-copy peptides were disabled. All experiments were performed on an HP Z2 SFF G9 Workstation equipped with an Intel^®^ Core™ i9-14900 processor and 64.0 GB of memory. Results showed that, for each dataset size, both tools produced identical sets of nonredundant protein sequences (Table 1, available as supplementary data at *Bioinformatics* online). Performance differences emerged only with larger datasets: for fewer than 10 000 genomes, runtime and memory consumption were comparable. However, beyond 25 000 genomes, WhatsGNU (Moustafa and Planet 2020) consumed on average 3.1 times more memory than BPSS but completed execution 1.42 times faster (Table 1, available as supplementary data at *Bioinformatics* online, Fig. 1). Linear regression analysis suggests that WhatsGNU (Moustafa and Planet 2020) would exceed 64 GB of memory consumption with >128 000 genomes, while BPSS would reach this threshold only beyond 622 000 genomes.

This workflow also allows users to rapidly obtain a set of peptide sequences specific to a protein of interest, enabling the detection of all its protein variability. To evaluate the accuracy of BPSS, we analyzed 41 *Staphylococcus aureus* proteins using a high-quality database constructed from 97 932 genomes (filtered from 100 000 assemblies; Table 1, available as supplementary data at *Bioinformatics* online). We calculated the proportion of genomes containing at least one variant of each protein and compared these values with literature data to determine whether each protein is part of the core or accessory genome. Among the 18 proteins considered as core, BPSS identified variants in an average of 97.44% of genomes [range: 93.33%–99.87%], aligning well with expectations. Lower detection rates for CoA and Spa likely reflect their high sequence variability, while the short length of PSMα peptides and frequent deletion-mutant of *hla* engineered by research laboratories, may also contribute to reduced detection (Table 1, available as supplementary data at *Bioinformatics* online). For these 41 virulence factors, BPSS ran in a median time of 14 min using a median of 3.2 GB of memory. Full results are available at https://sourceforge.net/projects/bpss/. We further assessed BPSS’s ability to select allelic variants by comparing it to blastp (Camacho *et al.* 2009). For this, we built a secondary database using amino acid sequences from five ESKAPE (Miller and Arias 2024) pathogens (excluding Klebsiella pneumoniae) with complete genome assemblies published between 2020 and 2025, excluding atypical and metagenome-derived genomes. The database included 660 genomes from *Acinetobacter baumannii*, 244 from *Enterococcus faecium*, 2999 from *Escherichia coli*, 863 from *Pseudomonas aeruginosa*, and 1159 from *Staphylococcus aureus* (Table 1, available as supplementary data at *Bioinformatics* online). We tested seven proteins: five conserved DnaA sequences, Spa from *S. aureus* (highly polymorphic), and GhxQ from *E. coli* (highly similar to GhxP). BPSS matched the performance of blastp overall, failing to retrieve only six of 645 sequences—five Spa variants and one DnaA variant—each significantly longer than the reference. Nonetheless, the final peptide outputs of BPSS encompassed these sequences, suggesting minimal impact on the overall detection of protein variability.

This pipeline was initially developed to enable targeted proteomics using selected peptide sequences to quantify protein expression across strains of a given species. However, beyond liquid chromatography–mass spectrometry applications, these peptides can also serve to detect proteins in genomic data. Additionally, several steps of the BPSS process produce intermediate outputs that support other applications, such as generating a nonredundant protein database with low memory usage and providing sequence statistics (e.g. composition, length, and per-strain distribution). The pipeline can also identify allelic variants of a target protein and report their frequency.

## Supplementary Material

btaf677_Supplementary_Data

## Data Availability

The latest release of BPSS and documentation can be found at https://gitbio.ens-lyon.fr/ciri/stapath/bpss or at 10.5281/zenodo.16894981. All containers are freely available on DockerHub platform at https://hub.docker.com/u/stapath.
